# Comparability and reliability of the positive and negative affect scales in the European Social Survey

**DOI:** 10.3389/fpsyg.2023.1034423

**Published:** 2023-03-22

**Authors:** Florencia M. Sortheix, Wiebke Weber

**Affiliations:** ^1^Research and Expertise Centre for Survey Methodology, Universitat Pompeu Fabra, Barcelona, Spain; ^2^Swedish School of Social Sciences, University of Helsinki, Helsinki, Finland

**Keywords:** cross-cultural equivalence, European Social Survey, positive affect, negative affect, reliability assessment

## Abstract

This study examined the measurement invariance of the positive and negative affect scales in the European Social Survey (ESS) in 2006 and 2012. We employed Multi-Group Confirmatory Factor Analysis with an estimator for ordinal data, allowing us to test threshold invariance, which had not been previously investigated for these scales. A 3-item measure of Positive Affect and a 5-item measure of Negative Affect showed that configural, threshold and metric (loading) and partial scalar (intercept) invariance held across almost all countries and between the two ESS Rounds. Our results provide cross-cultural validity to a broader measure of negative affect than past research using the ESS and examine these scales across more countries than any past study. Besides providing valuable insights for researchers interested in well-being and the ESS, our study also contributes to the ongoing discussion about diverging analytical choices in invariance testing.

## Introduction

In recent decades, self-reports of happiness and well-being have become accepted as a source of information used to inform public policy and compare countries [e.g., the World Happiness Report by Helliwell et al. (2019)]. Subjective well-being (SWB) is the most widely used construct to assess well-being and is defined as comprising a cognitive component, which refers to the evaluation of life satisfaction, and an affective component, including positive and negative affect, which refer to individuals’ report of positive, pleasant versus negative, unpleasant emotional states (Diener et al., 2003, 2018). Most of the research comparing the ranking of nations has done so based on average scores on a single aspect of subjective well-being, namely Diener’s Satisfaction with Life scale (SWLS; Diener et al., 1985) or single items assessing life satisfaction like the Cantrill ladder in the World Happiness Report (Helliwell et al., 2019). This information generally relies on single-item measures and lacks equivalence assessments to see whether these are comparable across countries (Oishi and Schimmack, 2010; Diener et al., 2013). It is crucial to examine the comparability of emotional well-being measures across cultures before making cross-country comparisons in large-scale surveys.

In this study, we used data from the European Social Survey (ESS) to analyse the cross-cultural equivalence of measures of Positive and Negative Affect and provide information on the comparability and reliability of these scales across countries. The ESS included twice the measures for Personal and Social Well-being developed by Huppert and So (2013) in Round 3 (2006) and Round 6 (2012). This rotating module contains 11 items assessing the frequency of positive (4 items) and negative (7 items) affective states in Rounds 3 and 6.

Our assessment is based on Multi-Group Confirmatory Factor Analysis to evaluate the comparability of scales across countries and time. The results will allow future researchers to use this scale and discard the possibility that cross-country and/or cross-time differences in the construct means or estimates of relationships (e.g., correlation or regression coefficients) reflect methodological artefacts rather than substantive differences by assessing measurement invariance (Davidov et al., 2018). Second, we provide researchers with the reliability or measurement quality of these scales to understand how well these scales measure the concepts of interest.

While there is agreement on the importance of testing for measurement invariance, the methodological approaches for conducting these tests are numerous (e.g., Davidov et al., 2018; Pokropek et al., 2019). Moreover, researchers need to make different analytical choices within each method, such as identification strategies, model testing, fit indices thresholds, or estimators. Therefore, we detail our selection of analytical strategies and specify how we aim to overcome the shortcomings of past research.

### Positive and negative affect

The organisation of affect has been described with various dimensions and structures, including Russell’s (1980) circumplex model based on the pleasant/unpleasant (valence) and the level of arousal of emotions, Thayer’s (1986) on the degree of energetic arousal, and Larsen and Diener’s (1992) eight combinations of pleasantness and activation. Watson and Tellegen (1985) proposed two dimensions of valence, originally named Positive and Negative Affect, but then changed to Positive and Negative Activation (Watson et al., 1999). The model depicted by Yik et al. (1999) incorporated all of these dimensions into an 8-dimension scheme. Later, Yik et al. (2011) proposed a 12-point circumplex of affect examined and validated in 33 societies with 25 different languages (Yik et al., 2022). The well-validated PANAS scale includes 20 items that tap into pleasant/activated emotions (e.g., alert, excited, interested) and unpleasant emotions (e.g., distressed, ashamed, nervous, irritable) validated in different samples (Lee et al., 2020). However, these extensive questionnaires cannot be included in large-scale surveys, and a short assessment of emotions was included in Rounds 3 and 6 of the ESS.

Huppert and So (2013) developed items to assess respondent’s affect based their items on other instruments such as the Positive and Negative Affect Scale (PANAS; Watson et al., 1988), the Scale of Positive and Negative Experience SPANE (Diener et al., 2010), the General Health Questionnaire (GHQ; Goldberg, 1981) or the Centre for Epidemiological Studies Depression scale (CES-D; Radloff, 1977). The CES-D is a crucial instrument in measuring depression and has been assessed in the ESS (Van de Velde et al., 2010). Depression, however, is conceptually different from NA and measures specific to NA have not been validated for the ESS data (for an exception for a short 3-item measure, Raudenská, 2020).

The ESS items do not tap into the specific dimensions of PANAS. Given the multi-dimensional structure of affect (Russell, 1980; Yik et al., 2011), we can categorise the items in the ESS as belonging to the following emotional dimensions based on Yik et al. (2011). For Positive Affect, one item taps into activated emotions (had a lot of energy), one item into pleasant/activated (enjoyed life), one item into pleasant (were happy), and one item into pleasant/deactivated emotion (felt calm and peaceful) dimensions. For Negative Affect, two items tap into unpleasant/deactivated (could not get going, felt that everything was an effort), three items into unpleasant (felt depressed, lonely, sad) and one into unpleasant/activated emotions (felt anxious).

Whether positive and negative affect are independent dimensions (e.g., Bradburn, 1969; Diener and Emmons, 1985) has generated some debate. In studies on the structure of affect, positive and negative affect have consistently emerged as two dominant and relatively independent dimensions. Diener and Emmons (1985) showed that the relationship between positive and negative affect differed significantly depending on the time frame. The correlation decreased linearly as time increased. Hence, they concluded that positive and negative affect states vary inversely but only over short periods; the two are likely to occur together within the same person at the same moment. For long time periods of weeks or more, the two types of affect become relatively independent: How much a person feels of one is unrelated to how much he or she feels of the other.

Given that ESS items tap into different dimensions on the pleasant-unpleasant, activated-deactivated dimensions, the ESS measures for Positive Affect (PA) and Negative Affect (NA) are not expected to be perfectly bipolar. The question in the ESS asked about emotions over a long period instead of in the moment or present/recent emotional states. The first step in our analyses was to analyse whether the items load on two different factors representing the concepts of PA and NA or whether there is no such distinction, and they load on one common factor.

### Measurement invariance of negative and positive affect scales

Some studies analysed the cross-cultural equivalence of measures of life satisfaction across countries (e.g., Vittersø et al., 2002; Oishi, 2006; Jang et al., 2017). Less research has analysed this for PA and NA scales. For a recent exception, Jovanović et al. (2021) presented equivalence tests for the Scale of Positive and Negative Experience in 13 countries but not the scales and countries included in the ESS. Some researchers have used items from the ESS to assess NA (depression; Sortheix and Schwartz, 2017) and positive and negative emotions (Kuppens et al., 2008), but have not examined their cross-cultural equivalence and have used a different set of items.

Two studies focused on the measurement of PA and NA using data from the European Social Survey (ESS)^1^. Fors and Kulin (2016) claimed measurement invariance for 2 two-item scales (one scale for each affect dimension), based on data from ESS Round 3, but did not detail the results or the invariance level achieved, nor did they test the data from ESS Round 6 or explained why he kept such a small number of items. In turn, Raudenská (2020), presenting a detailed explanation of the procedure, reached a final model with two reduced three-item scales for PA and NA and found approximate cross-country and cross-time scalar invariance in countries present in both Rounds 3 and 6. Raudenská (2020) used data from only the 21 countries present in both Rounds 3 and 6. Instead, we will use all available data and examine equivalence for all countries present in Round 3 (49 groups: 24 countries for PA, and 25 for NA); and R6 (57 groups, 29 for Positive Affect, and 28 for Negative Affect).

While Raudenská’s (2020) approach was adequate and she accounted for the ordinal nature of the items, the analyses ended up with a 3-item reduced measure for NA. This reduced measure included the item “sleep was restless” which is not generally included in categorisations of emotions (see, for instance, Yik et al., 2011 review). In sum, the analyses presented in this study include a larger number of countries compared to Raudenská (2020) and Fors and Kulin (2016) and allowed us to examine whether equivalence for more comprehensive scales for emotions would hold invariance using a different and superior methodology than before.

### Levels of invariance In this study

Configural equivalence means that the measurement model for the latent concept has the same factor structure across cultural groups. Configural equivalence means that the latent concepts can be meaningfully discussed in all countries and thus can be seen as pertaining to the category of interpretative equivalence definitions. Since configural equivalence is a prerequisite for further equivalence testing, it is often used as a baseline (Vandenberg and Lance, 2000). For examining threshold invariance, the threshold model considers ordered categories as a discretised version of a normally distributed latent continuous response through a number of threshold parameters. These thresholds indicate values of the continuous latent response where individuals cross over from one ordinal category to the next (Wu and Estabrook, 2016). This level of invariance tests that the thresholds are equal between groups. Loading (or metric) invariance, refer to the strength of the factor loadings, which can differ across countries. Loading equivalence (Baumgartner and Steenkamp, 1998) tests that factor loadings in the measurement model are invariant over groups.

However, configural, threshold, and loading equivalence do not yet lead to full-score comparability, as latent variable scores can still be uniformly biased upward or downward. An even stronger test for measurement equivalence is intercept (or scalar) equivalence. Within the MGCFA framework, intercept equivalence can be defined as the equality of intercept parameters over groups. This makes it possible to compare raw scores in a valid way, which is a prerequisite for country-mean comparisons. It makes no sense to compare groups with a scale that is not invariant since lack of invariance indicates that the correspondence between the true level of an attribute and the measure of that attribute differs across groups. In such a situation, one cannot know whether an observed difference is due to a real difference in the construct or differences in the interpretation of the response options attached to the scale. Researchers have argued that full scalar invariance (i.e., invariance of the parameters for all items), is not always necessary for meaningful group comparisons (Baumgartner and Steenkamp, 1998; Davidov et al., 2011). Provided that at least two items per latent construct; in this case, item 1 fixed to unity and one other, are equivalent, cross-national comparisons on latent means can be made.

Therefore, we aimed to keep the maximum amount of information and items in our models by fitting Confirmatory Factor Analysis models to establish partial invariance based on a local fit testing procedure to detect partially invariant items. Our analyses account for the ordinal nature of the items by using a model with threshold parameters, and we also tested the invariance of thresholds with a local fit testing procedure, something we are not currently aware of in any prior study. We follow the local fit testing procedure by Saris et al. (2009), which considers the modification indices, the size of the expected parameter change, and the power of the modification index test for the size of misspecifications. This approach provides reliable information on which parameters are misspecified.

This means that we separately tested four levels of invariance: configural invariance, threshold invariance, loading (also known as metric) invariance, and intercept (also known as scalar) invariance. Simulations by Pokropek et al. (2019) have shown the superiority of this approach: when partial invariance is present, the estimation of latent means of models based on approximate invariance with short scales (as done in Raudenská, 2020) or models that ignore partial invariance was worse than models that establish exact partial invariance. We also performed simultaneous cross-cultural and cross-time invariance tests, including all countries measured at each ESS round. The results determine the equivalence level of PA and NA in countries included in either Round 3 or Round 6 of the ESS, and longitudinally across the two waves. We further provided reliabilities of the equivalent scales analysed that allow scholars to evaluate the measurement quality of these scales and to correct for measurement errors.

## Methodology

### Participants

The data were drawn from the ESS Round 3 (R3) collected in 2006/2007 and Round 6 (R6) collected in 2012/2013 (European Social Survey, 2006, 2012). The ESS is a face-to-face survey designed to track the attitudes and behaviours of European citizens, which takes around 1 h. It consists of strict probability samples representative of the national population aged 15 years and older. Detailed information is available online at: https://www.europeansocialsurvey.org/. We used all available countries in Round 3 (24 for Positive Affect, *N* = 45,581; and 25 for Negative Affect, *N* = 47,099); and R6 (29 for Positive Affect, *N* = 54,673; and 28 for Negative Affect, *N* = 53,472). Differences in the number of countries are explained by the lack of data on one item in Hungary R3 and Albania R6.

To assess time invariance, we included countries present at both rounds. Twenty-one countries had data for Positive Affect and 22 for Negative Affect at R3 and R6, respectively. The ESS includes representative samples at each time point, but the participants are not the same, i.e., data is not longitudinal. Hence, time invariance can only be assessed at the country level.

### Measures

#### Negative affect

Six items asked how often in the week before the survey respondents had felt each of the following: felt depressed, felt that everything was an effort, felt anxious, felt lonely, felt sad, and could not get going. Four labelled response categories were offered: none or almost none of the time, some of the time, most of the time, and all or almost all of the time. The item “sleep was restless” was also present in both ESS Rounds as part of the same module. We excluded it from the beginning because we did not consider this item to tap an emotion.

#### Positive affect

Four items asked how often in the week before the survey respondents had felt each of the following: were happy, enjoyed life, had a lot of energy, and felt calm and peaceful. The response categories were the same as for Negative Affect.

### Analytical strategy for examining cross-country and cross-time equivalence

To determine our configural model, we performed Exploratory Factor Analyses (EFA), using R 4.0.4 (R Core Team, 2021) and the R package psych, version 2.0.12 (Revelle, 2021), with a weighted least squares estimator (WLSMV) based on polychoric correlations given the ordinal nature of the data. After that, we also tested the fit of the configural invariant model.

For model estimation, we used R 4.0.4 (R Core Team, 2021) and the R packages lavaan 0.6–8 (Rosseel, 2012) and semTools 0.5–4 (Jorgensen et al., 2018). Model syntax was created using measEq.syntax() function within SemTools, and we tested the local fit using the miPowerFit() function, which implements the procedure by Saris et al. (2009). The R codes used for the analyses and the EFA results can be found at: https://osf.io/k7sjh/files/osfstorage.

To test the invariance of the models, we used Multi-Group Confirmatory Factor Analysis (MGCFA). It is generally stated that ordinal variables with five or less categories cannot be treated as continuous (Li, 2016). Therefore, we used the WLSMV estimator (Flora and Curran, 2004; Brown, 2015; Li, 2016). This modelling approach determines the relation between the indicators and the latent factors by the thresholds, loadings, and intercepts. Configural invariance consists of fitting the same model across all groups without equality constraints on the parameters’ values. We sequentially added equality constraints on each parameter across groups.

For model identification, some constraints must be placed on the parameters, which are especially complex for ordinal data (Millsap and Yun-Tein, 2010; Wu and Estabrook, 2016). We adopted the approach introduced by Wu and Estabrook (2016). This follows the philosophy advocated by Schroeders and Gnambs (2020) of constraining only the minimum parameters required for identification at each level of invariance, so that invariance tests are not conflated with unneeded constraints on other parameters that make tests too stringent (e.g., on the variances’ size, as common in applied research, Schroeders and Gnambs, 2020).

We relied on local model fit, which allows testing whether each parameter is misspecified or not (Saris et al., 2009). Local fit testing aims to detect misspecified parameters in a given group (i.e., if an equality constraint on this parameter is incorrect) and is, therefore a suitable approach for detecting partially invariant items in each group. This approach differs from global fit testing, in which the models are accepted or rejected based on the value of statistics as the Chi-Square and/or the differences or absolute values of fit statistics [e.g., Comparative Fit Index (CFI), Root Mean Square Error of Approximation (RMSEA)]. These indices have been criticised for several reasons, such as sensitivity to sample sizes or unequal sensitivity to different model misspecifications (e.g., Saris et al., 2009). Global fit is also problematic in the context of invariance testing because models are usually accepted or rejected as a whole. This is unrealistic in settings with many groups: global fit indices are of little help for detecting partial invariance in specific parameters.

Therefore, we relied on local fit testing to detect partial invariance, but we provided global fit indices to establish the overall model fit. Following Saris et al. (2009), the criterion for the size of misspecifications to be detected is 0.2 for (unstandardised) thresholds and (standardised) 0.2 for correlated errors, 0.1 for loadings and 0.2 for intercepts. In each step, we determine first whether there is full invariance, i.e., no misspecified parameters according to local fit testing. When there is a misspecified parameter according to local fit testing, we freed it from the equality restriction and re-estimated the model. We repeated this process, changing parameters one by one until miPowerFit() did not suggest any more misspecified parameters. We then moved to the next level of invariance testing and repeated this process until we found no misspecifications.

Concerning reliability, past research using the ESS PA and NA items has primarily used Cronbach’s alpha to assess the quality of the measures. For example, the average Cronbach’s alpha across countries for the six items included in the ESS measuring NA was 0.81 (range 0.68–0.87; Sortheix and Schwartz, 2017). Kuppens et al. (2008) also reported within-country internal consistency reliabilities (Cronbach’s alpha) for the scale scores averaged 0.73 (SD = 0.08, range = 0.31) and 0.76 (SD = 0.05, range = 0.21) for positive and negative emotions, respectively. Here we present results based on the categorical omega developed by Green and Yang (2009), which does not assume tau-equivalence (i.e., all items in each group have equal loadings) and provides a reliability estimate of the sum scores of ordinal items which should not be treated as continuous (Flora, 2020). We extracted this coefficient for each group using the reliability() function within the semTools (Jorgensen et al., 2018) package, in R 4.0.4.

## Results

### Measurement model

First, we examined the factor structure of our measures using Exploratory Factor Analysis (EFA). We used a weighted least squares estimator based on polychoric correlations given the ordinal nature of the data. We examined eigenvalues and parallel analysis results which suggested a two-factor solution. Then, we compared the fit of EFA models with pre-defined with one and two-factor solutions. We used the GPArotation package in R with oblique rotation, which allows the factors to be correlated.

The loadings of the items for PA and NA are shown in Table 1. Table 2 presents results from the one- and two-factor solutions for Rounds 3 and 6. Considering the results from eigenvalues and parallel analyses, plus the fact that global fit indices improve from the one to two-factor solution, the latter appears to be the better model.

**Table 1:** Factor loadings for items measuring Positive and Negative Affect.

Exact wording: please tell me how much of the time during the past week…	Loading R3 Factor 1	Loading R3 Factor 2	Loading R6 Factor 1	Loading R6 Factor 2
*Positive Affect*				
…you were happy?	0	0.8	0.02	0.84
…you enjoyed life?	0.04	0.85	0.03	0.85
…you had a lot of energy?	−0.07	0.58	−0.09	0.59
…you felt calm and peaceful?*	−0.14	0.52	−0.19	0.53
*Negative Affect*				
…you felt depressed?	0.79	−0.06	0.77	−0.08
…you felt that everything you did was an effort?*	0.68	0	0.67	0
…you felt lonely?	0.64	−0.08	0.71	−0.01
…you felt sad?	0.86	0.04	0.87	0.03
…you could not get going?	0.68	−0.01	0.71	0
…you felt anxious?	0.74	0.05	0.72	0.03

^*^Items not included in the final model.

**Table 2:** Global fit indices for measurement models with one, two or three factors in each ESS round (*N* ~ 45,000) total number of observations was 54,673.

Round	*χ*^2^(*df*)	*p* value	TLI	RMSEA (90% CI)	RMSR	BIC
*ESS3*						
1-Factor	27,874 (35)	<0.000	0.81	0.142 (0.14/0.143)	0.08	32729.88
2-Factors	4202.13 (29)	<0.000	0.932	0.085 (0.083/0.086)	0.03	8501.87
*ESS6*						
1-Factor	28999.78 (35)	<0.000	0.805	0.149 (0.147/0.15)	0.08	41935.64
2-Factors	5503.74 (26)	<0.000	0.926	0.091 (0.09/0.093)	0.03	11625.92

*χ*^2^(df) = Chi-square (degrees of freedom), TLI = Tucker Lewis Index of factoring reliability, RMSEA (90% CI) = Root mean square error of approximation with 90% Confidence Intervals, RMSR = root mean square of the residuals.

As shown in Table 1, all items measuring negative emotions loaded on one factor (Negative Affect) and the remaining loaded on another factor (Positive Affect), with a latent factor correlation around − 0.7 in both rounds. Cross-loadings were non-existent or negligible for all items except for the item “felt calm and peaceful.” The cross-loading of this PA item with NA was − 0.14 in R3 and − 0.19 in R6. Even though it seems small for conventional analyses in such large samples, the presence of cross-loading in the model can lead to the misspecification of the latent model (Henseler et al., 2015). Since all other items reflect a pleasant and/or arousal emotion (Russell, 1980), and only this item (“felt calm.”) created issues for model estimation, we decided to exclude it from the measurement model.

We then proceeded to use Multi-Group Confirmatory Factor Analyses and tested whether the configural invariance of the model holds across countries. The Positive Affect (PA) model was just identified as it has only three items. Therefore, configural invariance testing was not possible, and we focused on correlated error terms when imposing threshold invariance. The local fit of the Negative Affect (NA) model suggested correlated error terms in eight groups, all involving the item “felt everything as an effort,” mostly correlated with the item “could not get going.” As we aimed to find a comparable model, we dropped the most problematic item, “felt everything as an effort.”

### Invariance testing

Table 3 shows the global fit indices of each specified model of invariance for PA. Results showed that threshold and loading (metric) invariance were held across all countries in both rounds (N = 53). In turn, full intercept (scalar) invariance did not meet the criteria, but partial intercept invariance held across all countries. The number of misspecified intercepts in the model was 33 with 0.2 criteria (See Electronic Supplementary Table S6 for the parameters).

**Table 3:** Global fit indices for different levels of measurement invariance of positive affect.

Invariance level	*χ* ^2^	Degrees of freedom	CFI	∆CFI	RMSEA	∆RMSEA
Configural	0	0	1	–	0	–
Thresholds	1631.920	156	0.994	−0.006	0.072	−0.072
Metric	2124.754	260	0.992	−0.002	0.063	−0.009
Scalar	10144.963	364	0.961	−0.031	0.121	0.058
Partial Scalar	4088.441	358	0.985	0.24	0.075	−0.046

The configural model was just identified.

Results of the measurement invariance tests for NA are presented in Table 4. For threshold invariance, we found misspecifications in the third threshold of some items for Russia in Rounds 3 and 6. Since misspecifications were only present in one threshold, we decided not to free by default loadings and intercepts for Russia in subsequent steps. Regarding loading invariance, local fit tests detected one non-invariant loading in Denmark (both rounds) and Finland (R3). We released the loadings for these items, thereby establishing full loading invariance for all countries but Denmark and Finland (i.e., for these two countries, only partial metric invariance was supported). The full intercept invariance model was not supported, but partial intercept invariance was established for all countries in both rounds (*N* = 53). The number of misspecified intercepts in the model was 60 with 0.2 criteria (See Electronic Supplementary Table S7 for the exact items).

**Table 4:** Global fit indices for different levels of measurement invariance of negative affect.

Invariance level	*χ*^2^	Degrees of freedom	CFI	ΔCFI	RMSEA	ΔRMSEA
Configural 1	6889.688	477	0.984	-	0.086	-
Configural 2	1382.134	265	0.997^†^	0.013	0.048	−0.038
Threshold	3118.636	525	0.992	−0.05	0.052	0.04
Partial threshold	2517.882	519	0.994	0.02	0.046†	−0.06
Loading	6214.129	728	0.983	−0.11	0.064	0.18
Partial loading	6124.899	727	0.984	0.01	0.064	0.00
Scalar	19858.99	934	0.943	−0.039	0.105	0.039
Partial scalar	5619.32	895	0.990	0.047	0.054	−0.051

Configural 1 refers to the model with the item “felt everything as an effort”; and Configural 2, to the model without the item.

**Figure 1:**
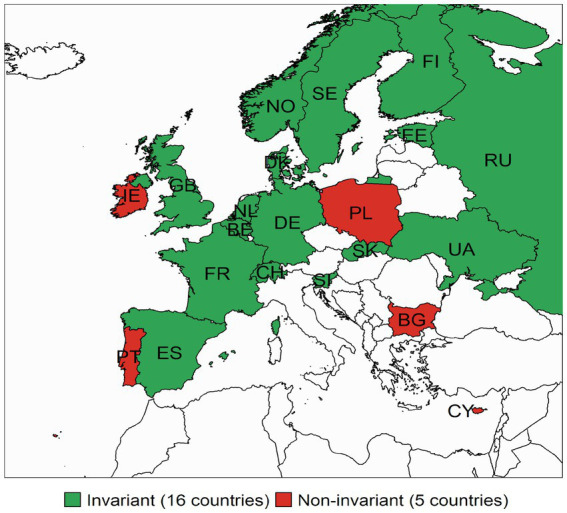
Countries with full intercept invariance on the measure of Positive Affect across time (Round 3–Round 6). The figure displays the countries where the full scalar invariant model holds across time points for countries present at both ESS rounds. The countries where PA was not fully invariant across time were Cyprus, Bulgaria, Poland, Ireland and Portugal.

**Figure 2:**
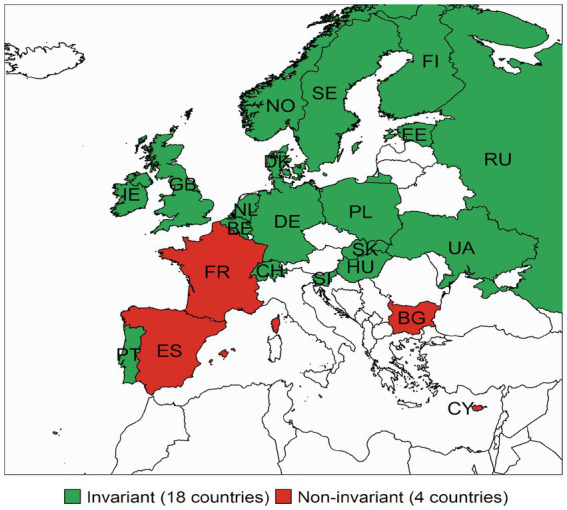
Countries with full intercept invariance in negative affect across time (Round 3–Round 6). The figure displays the countries where the full scalar invariant model holds across time points for those countries present at both ESS Rounds. The countries where NA was not fully invariant across time were Cyprus, Bulgaria, France, and Spain.

Lastly, we examined the *time invariance* for the scales of PA and NA among the 21 countries that were part of both ESS rounds. Figure 1 shows the countries where full intercept invariance models hold and those where the model did not hold. Importantly, partial intercept invariance was established across time for all countries. Results for NA are presented in Figure 2. Again, we present countries with full intercept invariance across rounds as the partial scalar invariance models across time were supported for all countries. We found over time full-intercept invariance (R3–R6) for the PA scale in 16 out of 21 (76%) of the countries present in both rounds. For NA this was satisfied in 18 out of 22 (82%) countries.

We also compared the difference between the latent means obtained by our models with the observed means. Results can be found in Electronic Supplementary material (Supplementary Tables S1–S4). Correlation between ranks of latent and observed means are 0.89 (Positive Affect, Round 3), 0.94 (Positive Affect, Round 6), 0.95 (Negative Affect, Round 3), 0.96 (Negative Affect, Round 6). Even though the correlation is relatively high, they are below desirable standards (Pokropek et al., 2019).

Supplementary Table S5 in Supplements shows the non-linear reliability estimate for each country in each round. Omega reliability scores for PA showed that in 11% of our samples, these were “good” (0.8 ≤ *q*^2^ < 0.9), in 81% were “acceptable” (0.7 ≤ *q*^2^ < 0.8), and in 8% were “questionable” (0.6 ≤ *q*^2^ < 0.7). Reliability for NA scale were in 45% of the samples “good,” in 53% were “acceptable,” and in 2% were “questionable.”

## Discussion

In this paper, we analysed the positive (PA), and negative affect (NA) measures included in two rounds of the European Social Survey, one of the most extensive surveys in the world. We provided an analysis for cross-cultural equivalence and reliability for a broader and conceptually grounded measure of NA than in past research (Raudenská, 2020) and validated the measures of PA and NA in more countries than ever before (Jovanović et al., 2021). We provide modelling information that allows future ESS users to use all available information and countries to research well-being using these scales.

Our results showed that threshold invariance and loading (or metric) invariance for our measure of PA (comprising three items: were happy, had a lot of energy, and enjoyed life) were present in all countries in R3 and R6. For NA (comprising five indicators: felt depressed, felt lonely, could not get going, felt anxious and felt sad), we found that threshold invariance holds for all countries but Russia, and loading invariance for all countries but Denmark (R3 and R6) and Finland (R3). These results are encouraging as they allow analysing correlations and regression for PA and NA (with the exceptions abovementioned) with other variables across almost all countries included in the ESS. Moreover, our results support the notion that threshold invariance is present, i.e., that respondents across countries, except Russia and Denmark, use the ordinal response scales here in similar ways.

Full intercept invariance was not present across countries, impeding mean comparison using observed or sum scores of measures of PA and NA in the ESS. The results from the *partial* intercept invariant models suggest that latent means can be compared across all groups (i.e., across countries and rounds). Moreover, the intercepts of these scales were invariant over time in most countries allowing for mean-change analyses for these countries. We found over time full-intercept invariance (R3–R6) for the PA scale in 16 countries and for NA in 18 of the 21 countries present in both rounds.

Important to note is that our initial measure for NA included the item “felt calm and peaceful” which was dropped due to cross-loadings (it loaded into the positive and negative affect latent factors in some countries) and created convergence issues. This item was not characterised by pleasant or activated affect as the other items (had a lot of energy, enjoyed life, and were happy), which are typical in positive affect scales (e.g., PANAS). Further studies could examine, more specifically, the role of pleasant/deactivated emotions in relation to measures of positive affect.

### Limitations

This possible flexibility in data analyses is analogous to what in psychology is called “researchers’ degrees of freedom” (Simmons et al., 2011) and can have substantial consequences for the conclusions reached. Comparing our results to the model of Positive Affect presented by Raudenská (2020), which used the same items as we did, the results are only partially convergent. For instance, we end up with different latent mean rankings of countries (Supplementary Tables S2 and S3). One possible reason for this variability is different analytical decisions, such as differences in testing procedures, the number of groups considered, estimators and concrete model specifications. Hence, based on the same data and aiming at measuring the same theoretical concept, different researchers can end up with different conclusions regarding scales’ composition or country ranks. These issues have been found in other fields and applications (e.g., Silberzahn et al., 2018). Thus, further research is needed to define the optimal strategy when differences in invariance tests and cross-cultural scale validation appear.

Furthermore, researchers have argued that measuring broad constructs with short scales is acceptable even if it comes with lower internal consistencies. This is because such scales take less time to complete while still capturing the depth of the construct (e.g., Diener et al., 2010). Multiverse analyses question this assumption and show that combining more items was associated with a smaller spread in correlation coefficients. Hence, shorter scales can increase the spread of the strength of association between constructs (Hanel and Zarzeczna, 2022). Another obvious limitation is that our contribution is limited to users of the European Social Survey, one of the world’s most extensive and most used surveys. The equivalence of our PA and NA measures for countries outside ESS would need further examination.

## Implications and conclusion

In conclusion, this paper provides valuable information on which scales to use for applied researchers. The results of the analyses showed that the ESS measure of positive (PA) and negative affect (NA) hold a two-factor structure of affect supporting original theoretical models with two dominant and relatively independent dimensions (e.g., Diener and Emmons, 1985). For the measurement of these constructs in the European Social Survey, we show that threshold invariance and loading (metric) invariance generally hold, allowing comparisons of the standardised relationships of the latent factor across countries. We also show that intercept (scalar) invariance is often not granted, which does not allow for comparing observed means. In order to overcome this, we provide scholars with two solutions: the latent means that can be compared across all countries, and we define subgroups of countries for which observed means can be compared across time (results presented in Figures 1, 2).

This contribution to the field allows other researchers to use this information when they study positive and negative affect as dependent variables or covariates. In addition, we also provide reliability estimates of the sum scores. Thus, thanks to the present study, researchers know how well the underlying concepts of interest are measured with the observed ESS data and can correct measurement errors (Saris and Revilla, 2016). Our overall findings have the practical implication that others do not need to invest in establishing measurement invariance or estimated measurement quality themselves.

## Data availability statement

Publicly available datasets were analyzed in this study. This data can be found at: https://www.europeansocialsurvey.org/data/.

## Author contributions

FS conceptualised and wrote the manuscript. WW edited and contributed to the manuscript, incorporated knowledge on scale development, and validated and guided the analyses. All authors contributed to the article and approved the submitted version.

## Funding

This research was funded by ESS ERIC as part of the Work Programme 1 June 2019–2031 May 2019. FS received a grant from the Emil Aaltonen Foundation nr. 170303 for postdoctoral research.

## Conflict of interest

The authors declare that the research was conducted in the absence of any commercial or financial relationships that could be construed as a potential conflict of interest.

## Publisher’s note

All claims expressed in this article are solely those of the authors and do not necessarily represent those of their affiliated organizations, or those of the publisher, the editors and the reviewers. Any product that may be evaluated in this article, or claim that may be made by its manufacturer, is not guaranteed or endorsed by the publisher.

## Footnotes

^1^Van de Velde et al. (2010) found partial scalar equivalence for the Depression scale of the Center for Epidemiologic Studies (CES-D; Radloff, 1977). This scale, however, uses 8 items (6 are negative and 2 are positive emotions) and represents a different construct.
