# Supplementary material for: Comparability and reliability of the positive and negative affect scales in the European Social Survey
Source: Front Psychol. 2023 Mar 22;14:1034423. doi: 10.3389/fpsyg.2023.1034423 (PMC10074487; doi:10.3389/fpsyg.2023.1034423)
Supplement: Supplementary file 1 [file Data_Sheet_1.docx]

- 1. **Electronic Supplementary Material**

**Table S1**

*Estimated Latent and Observed Means of Positive Affect and Rank Order in Round 3*

| Country | Latent mean | Observed means | Rank (latent means) | Rank (observed means) | Difference between ranks |
| --- | --- | --- | --- | --- | --- |
| Ireland | *0.92-[0.05]* | *2.9-[0.02]* | 1 | 2 | -1 |
| Switzerland | *0.84-[0.05]* | *2.96-[0.01]* | 2 | 1 | 1 |
| Belgium | *0.78-[0.05]* | *2.83-[0.02]* | 3 | 9 | -6 |
| Austria | *0.73-[0.05]* | *2.88-[0.01]* | 4 | 4 | 0 |
| Netherlands | *0.67-[0.05]* | *2.88-[0.01]* | 5 | 5 | 0 |
| United Kingdom | *0.65-[0.05]* | *2.76-[0.01]* | 6 | 10 | -4 |
| Slovenia | *0.64-[0.05]* | *2.89-[0.02]* | 7 | 3 | 4 |
| Cyprus | *0.63-[0.06]* | *2.68-[0.02]* | 8 | 17 | -9 |
| Sweden | *0.59-[0.05]* | *2.73-[0.02]* | 9 | 11 | -2 |
| Denmark | *0.57-[0.05]* | *2.88-[0.02]* | 10 | 6 | 4 |
| France | *0.53-[0.05]* | *2.86-[0.02]* | 11 | 8 | 3 |
| Norway | *0.53-[0.05]* | *2.87-[0.01]* | 12 | 7 | 5 |
| Slovakia | *0.43-[0.05]* | *2.73-[0.02]* | 13 | 12 | 1 |
| Spain | *0.42-[0.05]* | *2.69-[0.02]* | 14 | 15 | -1 |
| Germany | *0.35-[0.04]* | *2.71-[0.01]* | 15 | 14 | 1 |
| Estonia | *0.33-[0.05]* | *2.69-[0.02]* | 16 | 16 | 0 |
| Poland | *0.32-[0.05]* | *2.67-[0.02]* | 17 | 18 | -1 |
| Ukraine | *0.26-[0.05]* | *2.64-[0.02]* | 18 | 19 | -1 |
| Finland | *0.25-[0.04]* | *2.72-[0.01]* | 19 | 13 | 6 |
| Portugal | *0.19-[0.05]* | *2.59-[0.02]* | 20 | 20 | 0 |
| Russia | *0.12-[0.04]* | *2.58-[0.01]* | 21 | 21 | 0 |
| Bulgaria | *-0.07-[0.05]* | *2.45-[0.02]* | 22 | 24 | -2 |
| Latvia | *-0.1-[0.04]* | *2.51-[0.01]* | 23 | 22 | 1 |
| Romania | *-0.24-[0.04]* | *2.51-[0.02]* | 24 | 23 | 1 |

*Note.* Standard errors are shown in brackets.

**Table S2**

*Estimated Latent and Observed Means of Positive Affect and Rank Order in Round 6*

| Country | Latent mean | Observed Mean | Rank latent | Rank observed | Difference in ranks |
| --- | --- | --- | --- | --- | --- |
| Iceland | *1.04-[0.06]* | *2.98-[0.02]* | 1 | 3 | -2 |
| Switzerland | *1.03-[0.06]* | *3.06-[0.02]* | 2 | 1 | 1 |
| Slovenia | *0.93-[0.05]* | *3.05-[0.02]* | 3 | 2 | 1 |
| Sweden | *0.89-[0.05]* | *2.88-[0.02]* | 4 | 10 | -6 |
| Ireland | *0.88-[0.05]* | *2.97-[0.01]* | 5 | 5 | 0 |
| Netherlands | *0.86-[0.05]* | *2.96-[0.02]* | 6 | 6 | 0 |
| Belgium | *0.84-[0.05]* | *2.87-[0.01]* | 7 | 11 | -4 |
| United Kingdom | *0.74-[0.05]* | *2.81-[0.01]* | 8 | 14 | -6 |
| Norway | *0.72-[0.05]* | *2.98-[0.01]* | 9 | 4 | 5 |
| Cyprus | *0.69-[0.06]* | *2.91-[0.02]* | 10 | 9 | 1 |
| Czechia | *0.69-[0.05]* | *2.81-[0.02]* | 11 | 15 | -4 |
| France | *0.67-[0.05]* | *2.94-[0.02]* | 12 | 7 | 5 |
| Denmark | *0.63-[0.05]* | *2.92-[0.02]* | 13 | 8 | 5 |
| Germany | *0.61-[0.04]* | *2.84-[0.01]* | 14 | 13 | 1 |
| Poland | *0.58-[0.05]* | *2.86-[0.02]* | 15 | 12 | 3 |
| Slovakia | *0.58-[0.05]* | *2.81-[0.02]* | 16 | 16 | 0 |
| Ukraine | *0.52-[0.05]* | *2.79-[0.02]* | 17 | 18 | -1 |
| Israel | *0.47-[0.04]* | *2.77-[0.01]* | 18 | 19 | -1 |
| Estonia | *0.42-[0.04]* | *2.74-[0.01]* | 19 | 20 | -1 |
| Italy | *0.41-[0.06]* | *2.65-[0.02]* | 20 | 22 | -2 |
| Finland | *0.39-[0.04]* | *2.81-[0.01]* | 21 | 17 | 4 |
| Kosovo | *0.36-[0.05]* | *2.63-[0.02]* | 22 | 24 | -2 |
| Russia | *0.35-[0.04]* | *2.71-[0.01]* | 23 | 21 | 2 |
| Spain | *0.29-[0.05]* | *2.61-[0.02]* | 24 | 25 | -1 |
| Bulgaria | *0.26-[0.05]* | *2.59-[0.02]* | 25 | 26 | -1 |
| Portugal | *0.18-[0.05]* | *2.64-[0.02]* | 26 | 23 | 3 |
| Hungary | *0.04-[0.04]* | *2.58-[0.02]* | 27 | 27 | 0 |
| Lithuania | *0.03-[0.04]* | *2.52-[0.01]* | 28 | 28 | 0 |
| Albania | *0-[0]* | *2.5-[0.02]* | 29 | 29 | 0 |

*Note.* Standard errors are shown in brackets.

**Table S3**

*Estimated Latent and Observed Means of Negative Affect and Rank Order in Round 3*

| Country | Latent mean | Observed mean | Rank latent | Rank observed | Difference in rank |
| --- | --- | --- | --- | --- | --- |
| Ukraine | 0.93-[0.04] | 1.96-[0.02] | 1 | 1 | 0 |
| Latvia | 0.8-[0.03] | 1.79-[0.01] | 2 | 4 | -2 |
| Russia | 0.78-[0.04] | 1.86-[0.01] | 3 | 3 | 0 |
| Hungary | 0.77-[0.04] | 1.93-[0.02] | 4 | 2 | 2 |
| Portugal | 0.64-[0.03] | 1.79-[0.01] | 5 | 5 | 0 |
| Slovakia | 0.53-[0.03] | 1.73-[0.01] | 6 | 7 | -1 |
| Estonia | 0.43-[0.04] | 1.66-[0.01] | 7 | 9 | -2 |
| Romania | 0.43-[0.03] | 1.71-[0.01] | 8 | 8 | 0 |
| Bulgaria | 0.42-[0.04] | 1.76-[0.02] | 9 | 6 | 3 |
| Slovenia | 0.29-[0.04] | 1.53-[0.01] | 10 | 14 | -4 |
| France | 0.2-[0.04] | 1.56-[0.01] | 11 | 11 | 0 |
| Poland | 0.15-[0.04] | 1.65-[0.02] | 12 | 10 | 2 |
| Spain | 0.12-[0.04] | 1.5-[0.01] | 13 | 17 | -4 |
| Cyprus | 0.11-[0.04] | 1.5-[0.01] | 14 | 18 | -4 |
| Netherlands | 0.09-[0.03] | 1.56-[0.01] | 15 | 12 | 3 |
| United Kingdom | 0.04-[0.04] | 1.53-[0.01] | 16 | 15 | 1 |
| Austria | 0-[0] | 1.52-[0.01] | 17 | 16 | 1 |
| Switzerland | -0.05-[0.04] | 1.46-[0.01] | 18 | 20 | -2 |
| Belgium | -0.07-[0.04] | 1.55-[0.01] | 19 | 13 | 6 |
| Ireland | -0.07-[0.04] | 1.47-[0.01] | 20 | 19 | 1 |
| Germany | -0.14-[0.04] | 1.41-[0.01] | 21 | 21 | 0 |
| Sweden | -0.34-[0.05] | 1.4-[0.01] | 22 | 22 | 0 |
| Norway | -0.35-[0.05] | 1.31-[0.01] | 23 | 24 | -1 |
| Finland | -0.52-[0.05] | 1.37-[0.01] | 24 | 23 | 1 |
| Denmark | -0.68-[0.07] | 1.3-[0.01] | 25 | 25 | 0 |

*Note.* Standard errors are shown in brackets.

**Table S4**

*Estimated Latent and Observed Means of Negative Affect and Rank Order in Round 6*

| Country | Latent mean | Observed mean | Rank latent | Rank observed | Difference in ranks |
| --- | --- | --- | --- | --- | --- |
| Ukraine | 0.98-[0.04] | 1.97-[0.02] | 1 | 1 | 0 |
| Russia | 0.69-[0.04] | 1.81-[0.01] | 2 | 3 | -1 |
| Kosovo | 0.66-[0.04] | 1.72-[0.01] | 3 | 7 | -4 |
| Lithuania | 0.64-[0.03] | 1.74-[0.01] | 4 | 5 | -1 |
| Hungary | 0.62-[0.04] | 1.83-[0.01] | 5 | 2 | 3 |
| Czechia | 0.56-[0.04] | 1.73-[0.01] | 6 | 6 | 0 |
| Bulgaria | 0.52-[0.04] | 1.76-[0.01] | 7 | 4 | 3 |
| Slovakia | 0.5-[0.03] | 1.7-[0.01] | 8 | 8 | 0 |
| Italy | 0.35-[0.04] | 1.67-[0.02] | 9 | 9 | 0 |
| Estonia | 0.33-[0.03] | 1.63-[0.01] | 10 | 12 | -2 |
| Spain | 0.3-[0.04] | 1.57-[0.01] | 11 | 14 | -3 |
| Portugal | 0.29-[0.04] | 1.64-[0.01] | 12 | 10 | 2 |
| France | 0.25-[0.04] | 1.61-[0.01] | 13 | 13 | 0 |
| Cyprus | 0.21-[0.05] | 1.64-[0.02] | 14 | 11 | 3 |
| Netherlands | 0.03-[0.04] | 1.54-[0.01] | 15 | 17 | -2 |
| Israel | 0.01-[0.04] | 1.45-[0.01] | 16 | 20 | -4 |
| Switzerland | -0.05-[0.04] | 1.46-[0.01] | 17 | 19 | -2 |
| United Kingdom | -0.05-[0.04] | 1.51-[0.01] | 18 | 18 | 0 |
| Belgium | -0.06-[0.04] | 1.55-[0.01] | 19 | 15 | 4 |
| Slovenia | -0.15-[0.05] | 1.4-[0.01] | 20 | 22 | -2 |
| Poland | -0.17-[0.05] | 1.55-[0.01] | 21 | 16 | 5 |
| Ireland | -0.22-[0.04] | 1.44-[0.01] | 22 | 21 | 1 |
| Sweden | -0.33-[0.05] | 1.4-[0.01] | 23 | 23 | 0 |
| Germany | -0.34-[0.04] | 1.38-[0.01] | 24 | 25 | -1 |
| Iceland | -0.35-[0.06] | 1.4-[0.02] | 25 | 24 | 1 |
| Finland | -0.52-[0.05] | 1.35-[0.01] | 26 | 26 | 0 |
| Norway | -0.55-[0.06] | 1.28-[0.01] | 27 | 28 | -1 |
| Denmark | -0.75-[0.07] | 1.3-[0.01] | 28 | 27 | 1 |

*Note.* Standard errors are shown in brackets.

**Table S5**

*Categorical Omega of scales of Positive and Negative Affect, Rounds 3 and 6*

|  | **Positive Affect** | | **Negative Affect** | |
| --- | --- | --- | --- | --- |
| **Country** | **Round 3** | **Round 6** | **Round 3** | **Round 6** |
| Albania | - | 0.70 | - | - |
| Austria | 0.72 | - | 0.78 | - |
| Belgium | 0.73 | 0.71 | 0.80 | 0.77 |
| Bulgaria | 0.79 | 0.83 | 0.85 | 0.85 |
| Cyprus | 0.80 | 0.79 | 0.69 | 0.82 |
| Czechia | - | 0.77 | - | 0.86 |
| Denmark | 0.69 | 0.73 | 0.78 | 0.80 |
| Estonia | 0.76 | 0.78 | 0.76 | 0.80 |
| Finland | 0.76 | 0.75 | 0.73 | 0.75 |
| France | 0.75 | 0.75 | 0.78 | 0.75 |
| Germany | 0.72 | 0.72 | 0.79 | 0.78 |
| Hungary | - | 0.75 | 0.83 | 0.83 |
| Iceland | - | 0.76 | - | 0.78 |
| Ireland | 0.75 | 0.79 | 0.76 | 0.84 |
| Israel | - | 0.76 | - | 0.79 |
| Italy | - | 0.74 | - | 0.76 |
| Kosovo | - | 0.72 | - | 0.73 |
| Latvia | 0.71 | - | 0.75 | - |
| Lithuania | - | 0.82 | - | 0.82 |
| Netherlands | 0.76 | 0.76 | 0.74 | 0.74 |
| Norway | 0.69 | 0.67 | 0.78 | 0.80 |
| Poland | 0.78 | 0.79 | 0.84 | 0.84 |
| Portugal | 0.82 | 0.82 | 0.84 | 0.86 |
| Romania | 0.79 | - | 0.78 | - |
| Russia | 0.80 | 0.76 | 0.82 | 0.80 |
| Slovakia | 0.72 | 0.77 | 0.75 | 0.77 |
| Slovenia | 0.73 | 0.79 | 0.80 | 0.85 |
| Spain | 0.75 | 0.75 | 0.85 | 0.83 |
| Sweden | 0.77 | 0.78 | 0.80 | 0.78 |
| Switzerland | 0.69 | 0.75 | 0.75 | 0.75 |
| Ukraine | 0.74 | 0.77 | 0.79 | 0.81 |
| United Kingdom | 0.78 | 0.76 | 0.78 | 0.78 |

**Table S6. Unstandardized intercepts, loadings and thresholds of the final model for Positive Affect with partial intercept invariance.**

| Variable  (name in ESS data set) | **Enjoyed life**  **(enjlf)** | | **Happy**  **(wrhpp)** | | **Lot of energy**  **(enrglot)** | |
| --- | --- | --- | --- | --- | --- | --- |
|  | R3 | R6 | R3 | R6 | R3 | R6 |
| **Thresholds (SE)** | | | | | | |
| Threshold 1 | -1.07  (0.037) | | -1.27  (0.042) | | -1.00  (0.033) | |
| Threshold 2 | -0.020  (0.026) | | -0.106  (0.028) | | 0.068  (0.020) | |
| Threshold 3 | 1.08  (0.036) | | 1.20  (0.039) | | 1.22  (0.035) | |
| **Loadings (SE)** | | | | | | |
| Invariant in all groups | .734 (.014) | | .789  (.015) | | .563  (.016) | |
| **Intercepts (SE)** | | | | | | |
| Invariant in countries:  **Albania, Austria, Switzerland, Germany, Estonia, Netherlands, Russia, Slovenia, Slovakia, Ukraine, Israel, Lithuania** | 0 | 0 | 0 | 0 | 0 | 0 |
| **Countries with non-invariant intercepts (SE)** | | | | | | |
| Romania | Non-Inv | - | Inv | - | Non-Inv | - |
| **Denmark** | Non Inv | Non-Inv | Inv | Inv | Inv | Inv |
| **Finland** | Non-Inv | Non-Inv | Inv | Inv | Inv | Inv |
| **France** | Non Inv | Non-Inv | Inv | Inv | Inv | Inv |
| **Norway** | Non Inv | Non-Inv | Inv | Inv | Inv | Inv |
| Poland | Inv | Non-Inv | Inv | Inv | Inv | Inv |
| **Cyprus** | Non-Inv | Inv | Inv | Inv | Non-Inv | Non-Inv |
| **Czech Republic** | - | Non-Inv | - | Inv | - | Inv |
| Italy | - | Non-Inv | - | Inv | - | Inv |
| Kosovo | - | Non-Inv | - | Inv | - | Inv |
| **Spain** | Inv | Inv | Non-Inv | Non-Inv | Non-Inv | Non-Inv |
| Hungary | - | Inv | - | Non-Inv | - | Inv |
| Portugal | Inv | Inv | Inv | Non-Inv | Inv | Inv |
| Bulgaria | Inv | Inv | Inv | Non-Inv | Inv | Inv |
| **Latvia** | Inv | - | Inv | - | Non-Inv | - |
| **Belgium** | Inv | Inv | Inv | Inv | Non-Inv | Non-Inv |
| **Great Britain** | Inv | Inv | Inv | Inv | Non-Inv | Non-Inv |
| Ireland | Inv | Inv | Inv | Inv | Non-Inv | Inv |
| **Iceland** | - | Inv | - | Inv | - | Non Inv |
| **Sweden** | Inv | Inv | Inv | Inv | Non Inv | Non Inv |

Legend:, Inv.=invariant parameter, Non-Inv = non-invariant parameter, **bold = countries invariant across times**, SE indicated in brackets, “-“ indicates the country is not analyzed/present in that round.

**Table S7. Unstandardized intercepts, loadings and thresholds of the final model for Negative Affect with partial scalar invariance. Standard errors are shown in brackets.**

| Variable  (name in ESS data set) | **Depressed**  **(fltdpr)** | | | **Lonely**  **(fltlnl)** | | | | **Could not get going**  **(cldgng)** | | | | **Anxious**  **(fltanx)** | | | **Sad**  **(fltsd)** | | |  |
| --- | --- | --- | --- | --- | --- | --- | --- | --- | --- | --- | --- | --- | --- | --- | --- | --- | --- | --- |
|  | R3 | R6 | | R3 | | R6 | | R6 | R3 | | | R6 | | R3 | R3 | | R6 | |
| **Thresholds (SE)** | | | | | | | | | | | | | | |  | | |  |
| Threshold 1 | 0.37 (0.020) | | | 0.46 (0.017) | | | | 0.12(0.018) | | | | 0.23 (0.017) | | | 0.12(0.021) | | |  |
| Threshold 2 | 1.57 (0.036) | | | 1.31 (0.028) | | | | 1.40 (0.031) | | | | 1.25 (0.026) | | | 1.51 (0.034) | | |  |
| Threshold 3 | 2.20 (0.049) | | | 1.82 (0.037) | | | | 2.09 (0.044) | | | | 1.93 (0.038) | | | 2.12 (0.046) | | |  |
| Thresholds non-invariant | | | | | | | | | | | | | | | | | |  |
| Country: Russia |  | | |  | | | |  | | | |  | | |  | | |  |
| Threshold 3 | 2.77(0.081) | | | 2.05(0.055) | | | | 2.54(0.072) | | | | 2.24(0.060) | | | 2.64(0.073) | | |  |
| **Loadings (SE)** | | | | | | | | | | | | | | |  | | |  |
| Invariant all groups | 0.811(0.011) | | | 0.37(0.013) | | | | 0.720(0.012) | | | | 0.661(0.012) | | | 0.846(0.10) | | |  |
| Loadings not invariant in | | | | | | | | | | | | | | | | | |  |
| Countries: |  | | |  | | | |  | | | |  | | |  | | |  |
| **Denmark** | Inv | | | Inv | | | | 0.425(0.02) | | 0.425(0.02) | | Inv | | | Inv | | |  |
| Finland | Inv | | | Inv | | | | 0.425(0.02) | | Inv. | | Inv | | | Inv | | |  |
| **Intercepts (SE)** | | | | | | | | | | | | | | | | | |  |
| Invariant in countries: | *0* | | | 0 | | | | 0 | | | | 0 | | | 0 | | |  |
| **Countries with non-invariant intercepts** | | | | | | | | | | | | | | |  | | |  |
| **Slovenia** | Non-Inv | | Non-Inv | | Inv | | Inv | Inv | | | Inv | Non-Inv | Non-Inv | | Inv | Inv | |  |
| Romania | Non-Inv | | - | | Inv | | - | Inv | | | - | Non-Inv | - | | Inv | - | |  |
| **Ukraine** | Non-Inv | | Non-Inv | | Inv | | Inv | Inv | | | Inv | Inv | Inv | | Inv | Inv | |  |
| Island | - | | Non-Inv | | - | | Inv | - | | | Non-Inv | - | Inv | | - | Non-Inv) | |  |
| **Germany** | Non-Inv | | Non-Inv | | Inv | | Inv | Inv | | | Inv | Non-Inv | Non-Inv | | Inv | Inv | |  |
| **Belgium** | Non-Inv | | Non-Inv | | Inv | | Inv | Inv | | | Inv | Non-Inv | Non-Inv | | Inv | Inv | |  |
| **Hungary** | Non-Inv | | Non-Inv | | Inv | | Inv | Inv | | | Inv | Inv | Inv | | Inv | Inv | |  |
| **Poland** | Non-Inv | | Non-Inv | | Inv | | Inv | Inv | | | Inv | Inv | Inv | | Inv | Inv | |  |
| **Denmark** | Inv | | Inv | | Non-Inv | | Non-Inv | Non-Inv | | | Non-Inv | Non-Inv | Non-Inv | | Inv | Inv | |  |
| Spain | Inv | | Inv | | Inv | | Non Inv | Non Inv | | | Inv | Non-Inv | Non-Inv | | Inv | Inv | |  |
| Cyprus | Inv | | Inv | | Non Inv | | Inv | Non Inv | | | Non Inv | Inv | Non-Inv | | Inv | Inv | |  |
| France | Inv | | Inv | | Inv | | Inv | Non-Inv | | | Non-Inv | Inv | Non Inv | | Inv | Inv | |  |
| Czech Republic | - | | Inv | | - | | Inv | - | | | Non-Inv | - | Non-Inv | | - | Non Inv | |  |
| **Finland** | Inv | | Inv | | Inv | | Inv | Non Inv | | | Non Inv | Inv | Inv | | Inv | Inv | |  |
| Israel | - | | Inv | | - | | Inv | - | | | Non-Inv | - | Non-Inv | | - | Inv | |  |
| Bulgaria | Inv | | Inv | | Inv | | Inv | Inv | | | Non-Inv | Non-Inv | Non-Inv | | Inv | Inv | |  |
| **Switzerland** | Inv | | Inv | | Inv | | Inv | Non Inv | | | Non Inv | Non-Inv | Non-Inv | | Inv | Inv | |  |
| Latvia | Inv | | - | | Inv | | - | Non-Inv | | | - | Inv | - | | Inv | .- | |  |
| **Norway** | Inv | | Inv | | Inv | | Inv | Inv | | | Inv | Non-Inv | Non-Inv | | Inv | Inv | |  |
| Kosovo | - | | Inv | | - | | Inv | - | | | Inv | - | Non-Inv | | - | Inv | |  |
| Netherlands | Inv | | Inv | | Inv | | Inv | Inv | | | Inv | Non-Inv | Non-Inv | | Inv | Inv | |  |

Legend:, Inv.=invariant parameter, Non-Inv = non-invariant parameter, **bold = countries invariant across times**, SE indicated in brackets, “-“ indicates the country is not analyzed/present in that round.
